# Design of a Virtual Reality Interactive Training System for Public Health Emergency Preparedness for Major Emerging Infectious Diseases: Theory and Framework

**DOI:** 10.2196/29956

**Published:** 2021-12-14

**Authors:** Yue Luo, Mei Li, Jian Tang, JianLan Ren, Yu Zheng, XingLi Yu, LinRui Jiang, DingLin Fan, YanHua Chen

**Affiliations:** 1 School of Nursing Southwest Medical University Luzhou City China; 2 Operating Room The Affiliated Hospital of Southwest Medical University Luzhou City China; 3 Department of Anesthesiology The Affiliated Hospital of Southwest Medical University Luzhou City China; 4 Department of Rheumatism and Immunology The Affiliated Hospital of Southwest Medical University Luzhou City China; 5 Department of Cardiac Surgery The Affiliated Hospital of Southwest Medical University Luzhou City China; 6 Department of Nursing The Affiliated Hospital of Southwest Medical University Luzhou City China

**Keywords:** virtual reality, major emerging infectious diseases, emergency preparedness, interactive narrative theory, situated learning theory, human-computer interaction theory, emergency simulation, public health, training, VR, epidemic, medical education, education

## Abstract

**Background:**

Sufficient public health emergency preparedness (PHEP) is the key factor in effectively responding to and recovering from major emerging infectious diseases (MEIDs). However, in the face of MEIDs, PHEP is insufficient, so it is necessary to improve PHEP. The rapid development of virtual reality and human-computer interaction provides unprecedented opportunities for innovative educational methods.

**Objective:**

This study designed a virtual reality interactive training system (VRITS) to provide an effective path for improving PHEP in the context of MEIDs so that the public can effectively respond to and recover from MEIDs.

**Methods:**

This study used interactive narrative, situated learning and human-computer interaction theories as a theoretical framework to guide the design of the system. We used the literature research method and the Delphi method; consulted multidisciplinary experts, such as infectious diseases, disease control, psychology, and public health personnel, to determine the educational content framework; and set up an interdisciplinary team to construct an operating system framework for the VRITS.

**Results:**

We named the VRITS “People’s War Against Pandemic.” The educational content framework includes 20 knowledge, emotion, and behavior skills in 5 aspects (cooperating with prevention and control work, improving emergency response ability, guaranteeing supplies and equipment, preparing economic resources, and maintaining physical and mental health). The operating system framework includes virtual interactive training, knowledge corner, intelligent evaluation, and community forum modules, and the core module is the virtual interactive training module. In this module, users control virtual characters to move in various scenes, and then identify and analyze the controllability and harmfulness of the evolving pandemic and select the correct prevention and control strategy to avoid infecting themselves and others.

**Conclusions:**

The development and sharing of the multidisciplinary theoretical framework adopted by People’s War Against Pandemic can help us clarify the design ideas and assumptions of the VRITS; predict training results; understand the ability of training to change emergency knowledge, emergency emotion, and behavioral responses to MEIDs; and promote the development of more effective training systems based on virtual reality.

## Introduction

### Background

Since the 21st century, major emerging infectious diseases (MEIDs) have had a disastrous impact on humanity. In particular, the worldwide COVID-19 pandemic has infected more than 100 million people in more than 200 countries and regions, and the number of deaths has reached approximately 2.5 million [1]. It has led to the collapse of medical systems, the outbreak of economic crises, and social disorder in many countries [2]. It can be seen that MEIDs are highly destructive, unexpected, complex, and uncertain, and it is difficult for the public to effectively deal with them. This is related to poor public health emergency preparedness (PHEP), which is manifested in the lack of emergency knowledge and skills and the existence of negative emotional and behavioral responses [3-5]. During the COVID-19 pandemic, the public exposed problems such as inadequate scientific literacy, weak ability to select and judge information, and failure to actively cooperate with the pandemic prevention and control work [6-8]. Therefore, the public needs training to increase emergency preparedness and systematic theoretical training to acquire practical skills to prepare for potential risks. Compared with general training, systematic theoretical training enables training to be carried out in a predetermined framework and reflects the requirements in design elements to meet the needs of users [9].

Nelson et al [10] defined PHEP as “the capability of the public health and health care systems, communities, and individuals to prevent, protect against, quickly respond to, and recover from health emergencies, particularly those whose scale, timing, or unpredictability threatens to overwhelm routine capabilities.” With the increasing number of MEIDs, governments and public health departments are paying increasing attention to the study of emergency preparedness. Oppenheim et al [11] constructed a global national-level emergency preparedness index framework from the following 5 dimensions: public health infrastructure, physical infrastructure, institutional capacity, economic resources, and public health communication. In 2017, the European Center for Disease Prevention and Control developed a competency model for European Union public health professionals, including the ability of discovery and assessment, policy making, adaptation and implementation, coordination and communication, and emergency risk communication with the public [12]. However, these are studies conducted from a state or institutional level rather than from the public level. This study defines PHEP for MEIDs as the ability of the public to effectively respond to and recover from MEIDs, including the preparedness of emergency skills, legal compliance, economic estimation, avoiding secondary disasters, and physical and mental health.

Traditional emergency preparedness training mainly includes books, networks, classroom learning, and campus lectures [13]. In addition, emergency drills are usually an effective means to improve PHEP [14]. There are various forms of drills, including tabletop exercises, functional exercises, and full-scale exercises. A 10-week tabletop drill of hospital disaster preparedness from 9 participating hospitals showed that their ability of emergency response and treatment (disaster preparedness of equipment, risk communications, positive responses, and surge capacity) improved [15]. The systematic review showed that current drills are mostly aimed at public health professionals, and then the drills are extended to nonpublic health professionals, such as medical students, dentists, and dental health workers with special functional needs to serve the community [16]. Emergency drills for MEIDs need a lot of workforce and material resources. In fact, it is difficult for the public to get an opportunity to participate in such emergency drills, so they may face the challenge of implementation.

With the rapid development of mobile internet and virtual reality (VR) technology, the superiority of virtual interactive training resources is attracting increasing attention, which has a positive impact on medicine and health, education and teaching, engineering technology, etc [17-19]. Virtual interaction adopts the mode of the combination of VR and human-computer interaction and uses the computer to generate a 3D space composed of graphics so that users can visually feel immersed in the virtual environment and realize the information exchange process between human and computer through human-computer interaction. Virtual Police (ViPOL) [20] uses a multidisciplinary background to put theory into practice and develop a virtual training system. However, most of these training systems only describe theoretical knowledge but rarely explain their theoretical basis. This restricts the observation angle, the way of thinking, and the ability to understand effective mechanisms, which are important for the design of a comprehensive VRITS.

### Objectives

To address the public’s need to participate in emergency drills, we combined emergency preparedness training with virtual interaction to develop a VRITS for PHEP for MEIDs. Here, we introduced the educational content, theoretical, and operating system frameworks of the VRITS for emergency preparedness for MEIDs. The system is named People’s War Against Pandemic, aiming to provide the public with information on emergency knowledge and skills, emergency emotion, and behavioral responses to MEIDs in order to effectively respond to and recover from the pandemic.

## Methods

### Educational Content Framework

Through the method of literature review, we initially constructed an educational content framework for PHEP for MEIDs based on the global national-level emergency preparedness index framework [8], the 4-level response for public emergency and citizen health emergency literacy in China. Furthermore, the framework includes 6 first-level indices and 21 second-level indices. The 6 first-level indices involve cooperating with prevention and control work, improving emergency response ability, perfecting basic equipment, ensuring personal safety, preparing economic resources, and managing self-emotion. Then, we consulted 12 experts through the Delphi method to revise the framework, including 3 public health experts, 3 infectious diseases experts, 4 disease control experts, and 2 psychology experts. These experts had high theoretical knowledge and rich practical experience and carried authority and representativeness. In addition, these experts had a bachelor’s degree or above, an intermediate title or above, and 8 years of working experience or more. We sent consultation questionnaires to the qualified experts by email. Index screening was mainly based on the importance of each index evaluated by the experts and their specific suggestions, and the indices were screened by the arithmetic mean, the coefficient of variation (CV) of each index, and the Kendall coefficient of concordance (KCC). Importance was assigned on a scale of 1-5, ranging from very unimportant to very important. The retention criteria of the indices were that the arithmetic mean was greater than 3.50, the CV was less than 0.25, and the KCC of the second round was greater than that of the first round.

### Theoretical Framework

**Figure 1:**
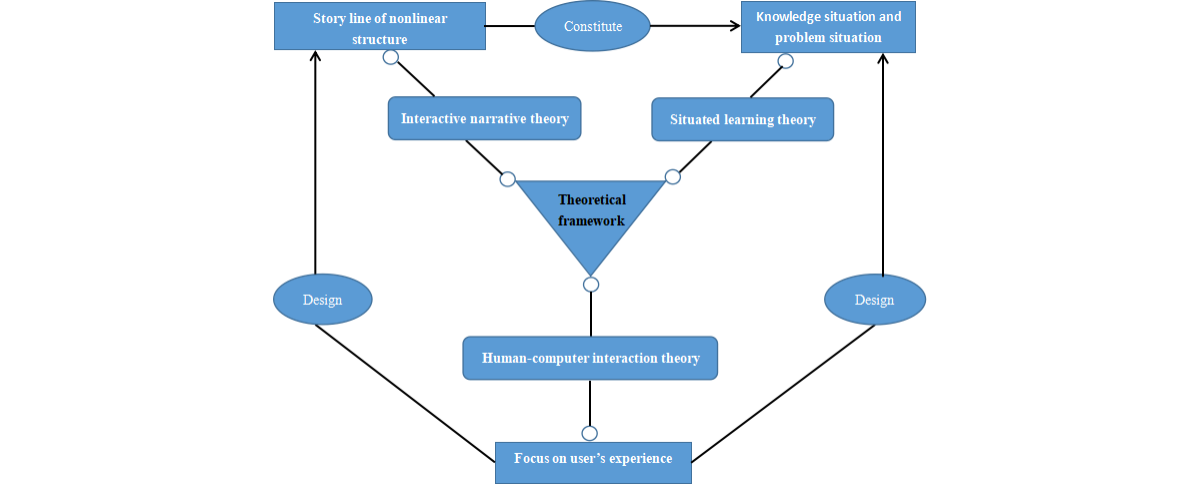
The theoretical framework.

People’s War Against Pandemic constructed the operating system using interactive narrative, situated learning, and human-computer interaction theories as a theoretical framework. Interactive narrative theory provides a reference for the design of a story line in the multidisciplinary background of a VRITS [21]. Situated learning theory provides a theoretical basis for the construction of an immersive, realistic, social, and practical environment [22]. Human-computer interaction theory centers on the user’s experience, which provides a theoretical basis for designing a user-friendly human-computer interaction interface [23]. In the design process of the VRITS, we integrated the 3 theories into a theoretical framework of the system (Figure 1). The story line that was designed with a nonlinear structure constituted the learning situation, and the human-computer interaction interface between the story line and the learning situation was designed with the users’ experience as the center.

#### Interactive Narrative Theory: Users Decide the Development of the Plot

Interactive narrative theory is a branch derived from narrative theory. It is a new narrative method that adds interaction on the basis of narrative. The characteristic of interactive narrative theory is that the development of the plot is decided by the users at the key nodes of the plot, and the small plots are connected to form a complete story in the process of interaction [24]. Compared with the traditional narrative mode of a single linear structure, interactive narrative is a nonlinear structure. Although the single linear narrative structure has branches, it eventually forms a 1D linear structure after simplification, which leads to the same result after each training session. Repetitive training can easily make the users tired, which is not conducive to effective absorption of knowledge. In the nonlinear narrative structure, the user is the controller of the whole process. At each interaction point, the user can randomly select an option, and the story and feedback change accordingly [25]. Interactive narrative pays more attention to the process of exploration. Even if the users fail, they can start again.

Based on the interactive narrative theory of a nonlinear structure, the plot of People’s War Against Pandemic was designed. The plot is about a MEID (COVID-19, cholera, Ebola, plague, or unknown-cause infection) outbreaking in a specific place. Users need to identify and analyze the controllability and harmfulness of the MEID, select the correct prevention and control strategies to control the virtual characters to move in various scenes (residential areas, medical institutions, pharmacies, police stations, banks, schools), and choose the correct behaviors to avoid infecting themselves and others. In this process, the users’ knowledge, emotions, and behavioral responses to prevention and control of the MEID are trained. After obtaining the users’ instruction information, the system creates multimodal interactive data to activate preset character scenes and synchronously generates matching dynamic pictures to form a complete training story.

In the whole training process, using the first-person perspective can increase the immersion of the users and trigger interaction nodes more conveniently. There are 5 main story lines for 5 kinds of infectious diseases, and each main story line has more than 5 interactive nodes. The first node is the selection of the type of infectious disease. Since the source of infection, transmission route, susceptible population, and harmfulness of each infectious disease are different, the choice of each node after the first node is different. Therefore, the choice of the first node is of vital importance. Then it leads to different plots with 4 interactive nodes of different scenes, prevention and control strategies, response measures, and personal situations. The users can improve their emergency preparedness in the process of making continuous choices. This highlights the causal relationship between decision making and results in the narrative process.

#### Situated Learning Theory: Learning in Real Situations

Situated learning theory holds that learning is not only an individual information acquisition process but also a social and practical participation process [26]. In the beginning, when situated learning theory was proposed, learners played roles in the real world. With the development of information technology, the environment of situated learning expanded to an online situation, simulation laboratories, and other virtual scenes [27], among which the VR interactive system is more immersive and realistic. In the emergency preparedness training in MEIDs, in view of the explosive and highly infectious characteristics of infectious diseases, users cannot learn in the real situation. Therefore, we designed a virtual learning situation based on the practical and social characteristics of situated learning, combined with the key nodes of interactive narrative stories.

The design of a virtual learning situation includes the design of a knowledge situation and a problem situation. A knowledge situation refers to the background and process situation in which knowledge occurs, which can help users construct the meaning of knowledge. A problem situation [28] emphasizes that learning should be placed in meaningful and complex situations so that users can take the initiative to learn and improve the ability to solve problems during the process of solving problems. We designed the knowledge and problem situations based on the nonlinear structured story line and the educational content for emergency preparedness. In total, 5 main story lines were designed with 5 complete learning situations, each with 5 branches of learning situations and 20 specific knowledge and problem situations.

#### Human-Computer Interaction Theory: Focus on Users’ Experience

Human-computer interaction refers to the process of information exchange between human and computer in a specific way with a specific language to achieve a specific function [29]. In the process of human-computer interaction, usability becomes important to accomplish users’ tasks efficiently, clearly, and safely. The usability criteria include a low learning cost, fast task execution, a low error rate, high satisfaction, and a high return visit rate [30]. Therefore, the design of human-computer interaction needs to focus on the users’ experience by taping their needs and analyzing their characteristics to make them have a good sense of experience.

To design an interactive interface that allows users to have a good sense of experience, we set up an interdisciplinary team of 10 experts from the fields of infectious diseases, education, psychology, and computers to repeatedly modify the preliminary design plan and interactive interface script and finally determine the design plan and script. The design plan is the function and realization path of each module of the operating system framework. The content of the script includes the interaction points involved in each interactive interface, all of which constitute the learning situation and story line. The preliminary design plan and script were modified and reviewed by experts from the fields of infectious diseases, education, and psychology and then handed to experts from the computer field to evaluate the implementation path of the interactive interface and preliminarily design several interactive interfaces. After the evaluation, the modification opinions were returned. Experts from the fields of infectious diseases, education, and psychology modified them. Experts from the computer field evaluated and again modified them until the final version was determined. In addition, before designing the VRITS, we conducted an investigation of the needs of more than 4000 Chinese students and found that in terms of training content, Chinese students have low awareness of infectious diseases and prevention and control skills [31]. Therefore, we paid more attention to the interface design of infectious disease knowledge and prevention and control skills.

## Results

### Educational Content Framework for PHEP for MEIDs

In the first round of expert consultation, the arithmetic mean of each first-level index was 4.33-4.92 and the CV was 0.06-0.15. In addition, the arithmetic mean of each second-level index was 4.08-4.92 and the CV was 0.06-0.34. According to the experts’ specific suggestions, the second-level indices assigned to “ensure personal safety” were more suitable to be assigned to other first-level indices, and the first-level index “ensure personal safety” was deleted. Therefore, the second-level indices in this deleted index were assigned to “cooperate with prevention and control work” and “improve emergency response ability.” In addition, some changes were made to other indices according to the experts' suggestions. Thus, 5 first-level indices and 20 second-level indices were formed, and a second round of expert consultation was conducted.

**Figure 2:**
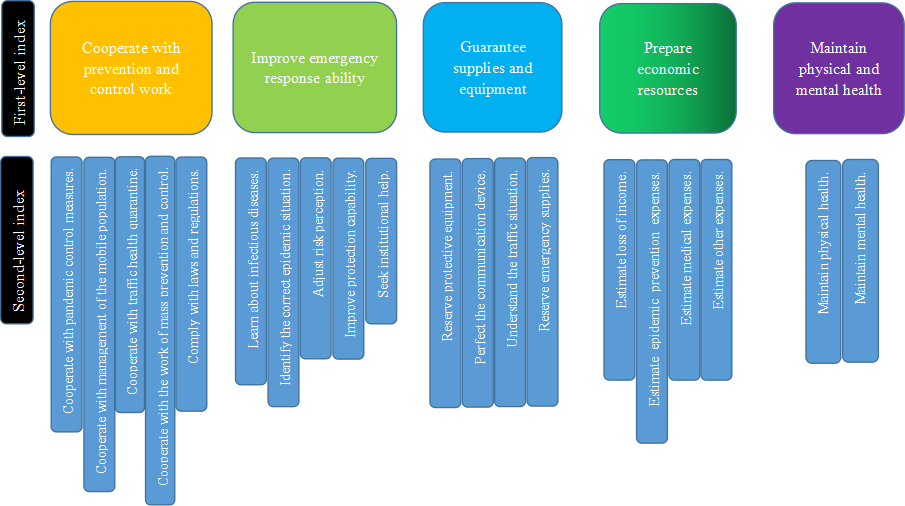
Educational content framework for PHEP under MEIDs. MEID: major emerging infectious disease; PHEP: public health emergency preparedness.

In the second round of consultation, the arithmetic mean of each first-level index was 4.42-4.92 and the CV was 0.06-0.12. In addition, the arithmetic mean of each second-level index was 3.67-4.92 and the CV was 0.06-0.22. According to the results of the second round of consultation, the scores of each index met the index retention criteria, and the KCC of the second round was greater than that of the first round (Table 1). The experts did not put forward specific opinions, so the index framework was not modified. Finally, the educational content framework for PHEP for MEIDs included 5 first-level indices and 20 second-level indices. The 5 first-level indices were “cooperating with prevention and control work,” “improving emergency response ability,” “guaranteeing supplies and equipment,” “preparing economic resources,” and “maintaining physical and mental health” (Figure 2).

**Table 1:** Kendall coefficient of concordance (KCC) for the 2 rounds of consultation.

Round		Entries, n	W	*χ^2^*	*P* value
**First round**					
	First-level index	6	0.258	15.474	.01
	Second-level index	21	0.269	64.448	<.001
	All indices	27	0.245	76.385	<.001
**Second round**					
	First-level index	5	0.270	12.952	.01
	Second-level index	20	0.358	81.626	<.001
	All indices	25	0.350	100.713	<.001

### Operating System Framework

**Figure 3:**
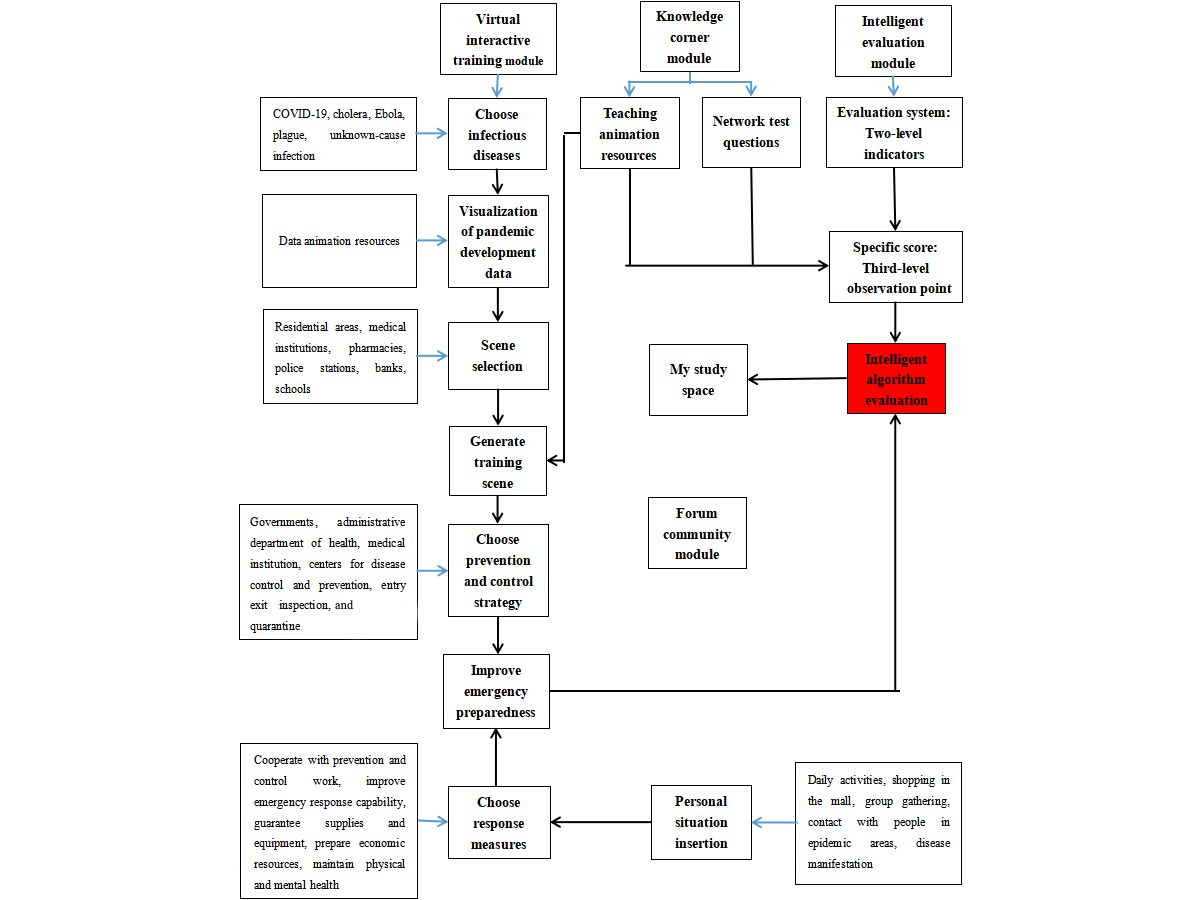
Operating system framework of the VRITS. VRITS: virtual reality interactive training system.

The operating system framework of the VRITS consists of 4 modules: virtual interactive training, knowledge corner, intelligent evaluation, and forum community. Figure 3 shows the overall design framework of the operating system. The development software we used was Unity 3d, the development tool was Microsoft Visual Studio, and the modeling tool was Autodesk 3ds Max. Users can access the system through a link on the web pages on computers, mobile phones, and tablets, and they can get it for free after registration.

#### Virtual Interactive Training Module

**Figure 4:**
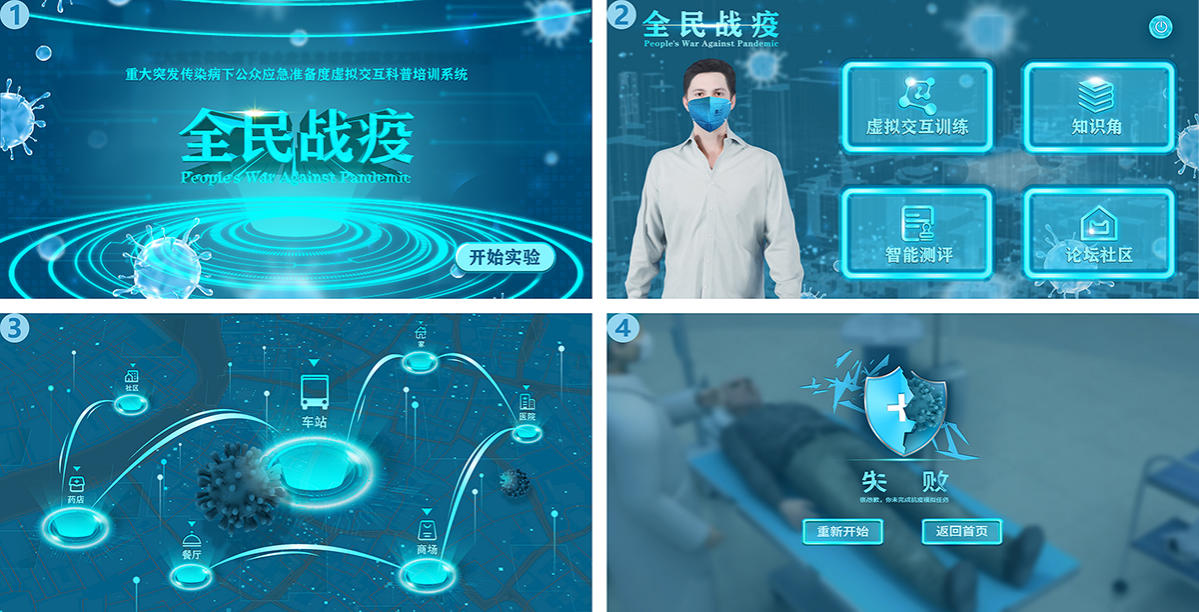
Interface of the VRITS. There are four interfaces in the picture. Interface 1 is the login interface; Interface 2 is the image of the virtual character and the entrance of the four modules; Interface 3 is the entrance of the different scenes; and Interface 4 is a warning interface for the virtual character infected with the disease after the training fails. VRITS: virtual reality interactive training system.

The characters in the virtual interactive training module are generated by computer (Figure 4). The users control the virtual characters to select specific prevention and control measures to complete the training tasks according to the visualized information about the pandemic development data and tasks prompted in the interactive interface. The virtual interactive training module is the core module of People’s War Against Pandemic, which contains 5 situations. The users randomly select a scene to enter, and multiple choices are set in the interactive nodes of each scene, including the selection of training routes, of answers to knowledge points, and of props in the props column. Every choice can get dynamic feedback. In the selection process, if a choice is wrong, the interactive interface prompts the users with an error and the score is deducted. The interactive text box of the interactive interface of each node prompts the users about the development of the pandemic, and a personal situation animation is inserted in the process of pandemic prevention, such as daily activities, shopping, group gathering, and contacting infected people in the pandemic area. If the users have corresponding clinical symptoms in their personal situation, they need to judge whether they are infected and then take corresponding countermeasures.

In the whole virtual interactive situation, if the virus is rampant and the pandemic spreads, a dialog box appears in the interactive interface to prompt the users to improve the level of prevention and control. Individuals should train their decision-making skills as well as their 20 knowledge and behavior skills in 5 aspects (cooperating with prevention and control work, improving emergency response ability, guaranteeing supplies and equipment, preparing economic resources, and maintaining physical and mental health). The success or failure of pandemic prevention and control is prompted by corresponding animation. If the user makes the right choices, they are not be infected and the interactive interface prompts “Training successful. Please check the score.” If the training fails, a warning appears on the interactive interface to prompt the virtual character to be infected with the disease (Figure 4). More seriously, it promotes the spread of the virus, the number of infected people rises sharply, and the pandemic cannot be controlled. Then the animation of secondary disasters corresponding to the diseases (crowd gathering, panic buying of supplies, shortage of medical resources) appears in the interactive interface as warning education.

#### Knowledge Corner Module

The knowledge corner module contains all kinds of knowledge related to MEIDs (including pathogeny, clinical manifestations, diagnosis, prevention and treatment). This knowledge is conveyed in the form of words, pictures, and videos, which is the supplement and integrity of the educational content of the virtual interactive training module. The administrator uploads the latest knowledge of infectious diseases (including the protection skills of infectious diseases, laws and regulations related to MEIDs, and secondary disasters caused by the pandemic) from the background of the system, and each knowledge point matches the network test questions and answers, which is convenient for users to test and then correct the answers.

#### Intelligent Evaluation Module

The intelligent evaluation module takes the indices at all levels of the educational content framework for emergency preparedness as specific scoring points. Every behavior of the users in the training process is recorded and intelligently scored. The training starts out on a 100-point scale, with points deducted in real time if the users make an incorrect choice. At the end of the training, the users can view the total score and the score for each first-level index. Automatic interactive text analysis technology, such as participation analysis, social network analysis, and content analysis, is used to obtain the users’ participation in learning, social network, concerned learning content, and online behavior information to analyze their characteristics and to push their personalized training resources. The module can also provide data collection, integration, classification, and application functions and offer objective and real-time big data for the VR interactive training in MEIDs.

#### Forum Community Module

In the forum community module, users can enter the forum community to participate in questions, discussions, and communications. They can publish topic posts related to the pandemic, help posts for difficulties encountered in simulation training, experience posts for success in fighting the pandemic, and announcement posts issued by administrators. Using the interactive, direct, and group characteristics of the forum, we can realize the interaction between user and user, user and interactive interface, and user and teaching content. This kind of real-time communication makes it easy to find and solve problems in time.

## Discussion

### Principal Findings

We drew knowledge from multiple disciplines, such as education, medicine, psychology, public health, and computers, which provided reference information for the theoretical framework design of People’s War Against Pandemic. Then we put forward the theoretical basis of multidisciplinary integration of interactive narrative, situated learning, and human-computer interaction and designed a VRITS. A clear theoretical basis can help us clarify the design ideas and assumptions of the VRITS; predict training results; understand the way of training to change emergency knowledge, emergency emotion, and behavioral responses to MEIDs; and promote the development of a more effective VRITS.

Whether interactive narrative theory plays a core role or a peripheral role in a VRITS usually depends on the teaching objectives of virtual interaction and the consequent changes in knowledge, emotion, and behavior. In the prevention and treatment of some chronic diseases and mental diseases based on virtual interactive games, interactive narrative often plays a secondary incentive role [32]. The changes in the symptoms of chronic diseases and mental state are usually rooted in daily life. In People’s War Against Pandemic, the users' knowledge and skills, emotional adjustment, and behavioral responses of emergency preparedness for MEIDs run through the main line of the whole interactive narrative, making it the core position in People’s War Against Pandemic. Therefore, users transform the knowledge and skills learned from virtual interaction into specific attitudes or behavioral responses in life, which is closely related to the role of the interactive narrative. In addition, how to deal with the relationship between story and interaction in interactive narrative is also particularly important [24]. In the process of virtual interactive training, there are many branches in each interactive node, and the choice of each branch has an impact on the final result. Eventually, each branch of all interactive nodes is arranged and combined into different stories. Each interaction determines the direction of the story, and the story is composed of every interaction.

Situated learning theory also plays an important role in a VRITS. The purpose of a virtual learning situation is to create a social and participatory experience for the users and realize the functions that cannot be completed in real life [33]. It is the cornerstone of the system. In the past few years, more fields have applied situated learning theory to design virtual learning situations. “Tawaf” [34] emphasizes the positive role of situated learning in improving learners' interest in learning by solving problems in a 3D VR environment with virtual characters. A study on a virtual learning environment for disaster risk reduction explored the factors that affect the sense of reality of a learning situation in a virtual environment [35]. Among them, the positive factors to pay attention to are socialization and learning from mistakes, which give learners a sense of reality in face-to-face interaction and to safely fail in a virtual environment. In brief, the use of a virtual learning situation can enhance the reality of the scene, the immersion of training, and the sense of experience and stimulate the motivation and efficiency of learning. In addition, situated learning and interactive narrative are integrated. The learning situation is composed of story lines with a nonlinear structure, and all stories take place in the learning situation, which is the carrier of the story lines.

If interactive narrative theory is the core of a VRITS and situated learning theory is the cornerstone of the VRITS, then human-computer interaction theory plays the role of a bridge of information exchange between the system and the users. According to the users’ cognitive ability and information acquisition ability, the human-computer interaction interface is designed centering on the users’ experience and the information communication between human and computer is finally realized [36]. Eventually, based on human-computer interaction theory, this study designed the interactive interface of a learning situation and story line, which organically integrated the 3 theories to make the information communication smooth and efficient.

The PHEP education for MEIDs mainly includes popular science knowledge, protection skills, and cooperation in pandemic prevention and control [37]. In addition, emergency preparedness, such as social cooperation, legal compliance, economic prediction, social stability, and avoiding of secondary disasters, are also important. Therefore, this study constructed an educational content framework of emergency preparedness to train users on 20 items of emergency knowledge and skills, emotion, and behavioral responses in 5 aspects. The findings will enrich the theoretical system of emergency preparedness education for MEIDs and provide the public with a panoramic understanding of the response to MEIDs so that their comprehensive preparation can be promoted and ultimately an effective response can be achieved.

As a new training system, People’s War Against Pandemic not only has the characteristics of a 3D stereoscopic combination of sound, text, image, and interaction but also uses a sense of reality of VR scenes to fully arouse the interest of users and satisfy their curiosity and thirst for knowledge. Through People’s War Against Pandemic simulation training, users can keep in mind the correct process and knowledge points of prevention and control of MEIDs in constantly wrong choices and effectively improve their ability to comprehensively apply knowledge and skills to improve emergency preparedness and reduce disease infection. At the same time, this training allows users to place themselves in a virtual situation from a first-person perspective, mobilizing the users’ subjective initiative and stimulating their learning motivation through role-playing and a realistic virtual environment.

As an innovative way of emergency training for infectious diseases, People’s War Against Pandemic is an attempt to develop emergency training resources for MEIDs. Its design of educational content is more comprehensive, covering many core parts of knowledge and skills related to MEIDs. In addition, the system can judge whether the prevention and control strategies for infectious diseases selected by users are correct behaviors, and timely feedback and adjustment of problems in the training process will play a substantial positive role in improving the learning effect [38]. Based on this, People’s War Against Pandemic has set up a real-time feedback function. The intelligent evaluation module records and evaluates each option of each user and presents the results in the interactive interface in the form of a score, which effectively realizes the real-time feedback of information. Offline emergency drills often face implementation challenges due to the cost of workforce, material, and financial resources. The online resources of People's War against Pandemic can be promoted to the public through the internet to solve the bottleneck problem of offline emergency drills. The system can be released by the municipal Center for Disease Control and Prevention and tried out among community residents. In the process of using the system, the residents' use of experience, satisfaction, opinions, and suggestions can be collected to modify and improve the system, and finally it can be promoted and applied nationwide.

### Limitations

There are some limitations in this study. The immersion of the VR interactive system designed in this study may make some young or low-self-control users indulge in it. However, this problem may be solved by limiting the length of training [39]. In addition, it will take some time to popularize the VR interactive devices. Some users may experience the effect of the VR interactive system on mobile software, but they cannot experience a more realistic sense of operation with VR devices. Moreover, users cannot customize roles for the time being. At present, the system roles are unified as citizens, and there is no division by age, gender, and occupation. Therefore, it may have some impact on users' immersion and usability. This problem can be solved when the system is upgraded and improved in the future. Most importantly, we did not conduct a randomized controlled trial on the effectiveness of VR interactive systems. Therefore, in the future, we need to carry out empirical research to verify the effectiveness of virtual interaction systems.

### Conclusions

A VRITS is a product of a highly integrated multidisciplinary background. The application of virtual interaction to the emergency preparedness training in MEIDs may solve the bottleneck problem in the current emergency preparedness training in MEIDs. Based on a solid theoretical foundation, rich teaching resources, a virtual learning situation, a complete story line, and an interactive function, the system realizes the visualization of emergency preparedness training for MEIDs, which may stimulate the learning motivation of users, increase their sense of experience and immersion, enhance their learning participation and learning efficiency, and improve the training effect. In future practice, to improve the level of PHEP for MEIDs, it is necessary to further perfect the VRITS and promote its use for emergency preparedness education of MEIDs.

## Abbreviations

CV: coefficient of variation
KCC: Kendall coefficient of concordance
MEID: major emerging infectious disease
PHEP: public health emergency preparedness
VR: virtual reality
VRITS: virtual reality interactive training system
